# Editorial: The paradigm of creating a new environment for travel industry consumers during the crisis and instability in the world

**DOI:** 10.3389/fpsyg.2022.1074451

**Published:** 2022-11-11

**Authors:** Tamara Gajić, Marko D. Petrović, James Kennell

**Affiliations:** ^1^Geographical Institute “Jovan Cvijić” Serbian Academy of Sciences and Arts (SASA), Belgrade, Serbia; ^2^Institute of Sports, Tourism and Service, South Ural State University, Chelyabinsk, Russia; ^3^Faculty of Hotel Management and Tourism, University of Kragujevac, Kragujevac, Serbia; ^4^School of Hospitality and Tourism Management, University of Surrey, Guildford, United Kingdom

**Keywords:** travel industry, consumers, environment, crisis, tourism

The humanity of the twenty-first century is faced with numerous natural and social disasters, which leave great and long-lasting consequences on the entire society and economy. Turbulence caused by the COVID-19 pandemic is still felt in the tourism sector today, and it is uncertain whether the invisible enemy of humanity is only in a period of lull, or whether it will completely withdraw. The goal of this Research Topic was to fully explore the emerging key problems in this newly created environment, both for businesses in a wide range of tourism activities, and for tourists themselves. The crisis situation has led to significant changes in the economic sphere, but also in the sphere of human interaction, reaction, and behavior. The results of the research on the given Research Topic pointed to all the visible consequences of the pandemic that the tourism industry and its related activities are facing, but the authors tried to identify the given problems to devise and propose strategic measures that would be applied in the recovery of the entire sector.

In their Research Topic, the authors Liu et al., point out that the need for health tourism is not so clearly expressed in the minds of Chinese tourists, regardless of the enormous consequences caused by the pandemic. China is a large market with a rich resource base, and the authors suggest the implementation of concrete measures with the support of the government sector for greater development of health tourism.

Calder et al. investigated the impact of COVID-19 on the behavior of pro-environmental travelers and the intention to travel to other countries for recreational reasons, using the health belief model (HBM) and the theory of planned behavior (TPB) for determining the factors that will influence the behavior of pro-environmental tourists after the pandemic. Certain categories of tourists and potential travelers are in favor of choosing ecological or green services, although this is not necessarily the only choice for travel. They found that fears regarding safety, as well as factors of the TPB model, have a positive influence on behavioral intention.

Zhu et al. claim that heritage tourism was also damaged during the pandemic. Uncertainty and new challenges are certainly a problem that needs to be faced, but closures during the crisis situation also caused the closure of cultural and heritage facilities for security reasons. They pointed out that emotions and personal attitudes are the drivers of personal norms and intentions, and the selection of cultural heritage is influenced by these drivers. Also using the TPB model and the extended form of the NAM model, they indicated that social pressure does not greatly affect tourists' intentions. This further implies the conclusion that the feeling of guilt due to non-compliance with security measures that have been established does not affect visits to cultural heritage sites.

Ecotourism was also was researched by Mengkebayaer et al. The authors proposed ways of researching and presenting attributes that serve to brand an ecotourism destination. They presented data that confirm the fact that the perceived value and experience gained in the tourist movement influence the creation of loyalty toward the tourist destination. Memory is a moderator in the relationship between destination attachment, destination equity, and destination loyalty. While destination attachment and destination equity are mediators of the relationship between perceived value, experience, and loyalty.

Xu et al. examine whether the measures of the absence of customs duties in certain countries can influence the intention of tourists to visit those countries during the pandemic. On a sample of 410 respondents, the obtained results were analyzed through Causal stepwise regression and bootstrap sampling analyses. The results confirm that destinations with duty-free travel are more attractive for tourists, and explain the counterfactual situation by pointing out that tourists in a state of less fear of the pandemic were more likely to visit such countries, and vice versa.

Aware of the overall damage caused by the pandemic, the authors Zhao et al. investigated with a large number of respondents the extent to which tourists are willing to share a short video, thus untangling previous mediators of parasocial relations between tourists and Vlogger videos. They showed that, to the greatest extent, the parasocial relationships of tourists and videos from the domain of tourism are influenced by motivation for entertainment, loyalty to values and emotional engagement.

Aleksić et al. conducted a study on airline choice and passenger loyalty during a pandemic. Using regression analysis, they determined that food quality and safety factors significantly influence attitude, subjective norms, and perceived behavioral control, in the case of medium- and long-haul flights. Intentions serve as a mediator in the relationship between: food quality, food and beverage safety, subjective norms, perceived behavioral control, and airline choice.

A Research Topic that is inevitable during the pandemic is the possession of a vaccination certificate, which is always a taboo topic and creates great limitations because very often in research respondents are given socially desirable responses. Gajić et al. investigated the predictive power of the Big Five (OCEAN) factors on the decision to visit restaurants during the pandemic, depending on having a certificate. The obtained results indicate the behavior of certain consumer profiles. Respondents with pronounced traits of openness and extraversion had positive attitudes and were ready to visit restaurants during the pandemic. Respondents with pronounced traits of conscientiousness and neuroticism had negative attitudes toward restaurant visits during the pandemic. However, the variable of possessing a vaccination certificate partially moderated the attitude of respondents with pronounced neurotic traits. Having a certificate partially reduces the fear of staying in closed facilities during the pandemic.

Akhrani et al. also emphasized the Research Topic of vaccines during the pandemic and the fear of it in their research. They highlight the different policies of the Indonesian and Taiwanese authorities in the fight against the pandemic compared to other parts of the world. They analyzed the travel intention of Indonesian and Taiwanese tourists, investigating the perception of pandemic risk, pandemic fear, vaccination attitude, and general fear of travel during that period. The results indicated that among Indonesian tourists, the intention to travel is influenced by all the mentioned factors directly and indirectly, while among Taiwanese tourists, the fear of COVID-19 is the only one that does not play a role.

Kürüm Varolgüneş et al. investigated how to create indicators of sustainable rural development in the course of increased tourist movements during the pandemic. The research highlights the process of creating awareness among local tourists about the sustainability of the destination and its resources. Local tourists are to the greatest extent the users of the rural tourism product during the pandemic. In their study, they used A'VOT and TOWS hybrid methods. They believe that by determining sustainability indicators, it will be possible to establish strategies for building strengths and eliminating weaknesses.

The importance of the Research Topic is reflected in the overview of the wide range of negative consequences caused by the pandemic in all parts of the world. The authors were given the freedom to, through the topic of the Research Topic, summarize the results of all the negative consequences experienced by the destination, but also to propose corrective measures for future operations. Based on the research presented in the special edition, it is possible to see all the difficulties that the tourism industry was facing, which includes the users of tourism services. Identifying key weaknesses and problems that can be caused by a crisis situation is of great importance in the application sense for future business in conditions that enable the safety of both service providers and tourists. However, the results of all the research presented will certainly have significance in a theoretical context in order to complement the existing literature and knowledge about the consequences of the pandemic. The fact is that every country and every form of tourism faced the crisis in different ways. The results of the research will remain as a witness to a difficult time in today's civilization. The importance is reflected in summarizing as much research as possible that would be available to science, economy and society. Considering the results, proposals for recovery and economic measures stability, this Research Topic will certainly contribute to future precaution and timely reaction to some future possible crisis situations.

## Author contributions

All authors listed have made a substantial, direct, and intellectual contribution to the work and approved it for publication.

## Conflict of interest

The authors declare that the research was conducted in the absence of any commercial or financial relationships that could be construed as a potential conflict of interest.

## Publisher's note

All claims expressed in this article are solely those of the authors and do not necessarily represent those of their affiliated organizations, or those of the publisher, the editors and the reviewers. Any product that may be evaluated in this article, or claim that may be made by its manufacturer, is not guaranteed or endorsed by the publisher.

